# NRG4-ErbB4 signaling represses proinflammatory macrophage activity

**DOI:** 10.1152/ajpgi.00296.2020

**Published:** 2021-04-07

**Authors:** Michael A. Schumacher, Isabella C. Dennis, Cambrian Y. Liu, Cache Robinson, Judie Shang, Jessica K. Bernard, M. Kay Washington, D. Brent Polk, Mark R. Frey

**Affiliations:** ^1^The Saban Research Institute, Children’s Hospital Los Angeles, Los Angeles, California; ^2^Department of Pediatrics, Keck School of Medicine, University of Southern California, Los Angeles, California; ^3^Department of Biochemistry and Molecular Biology, Keck School of Medicine, University of Southern California, Los Angeles, California; ^4^Department of Pathology, Microbiology, and Immunology, Vanderbilt Ingram Cancer Center, Vanderbilt University Medical Center, Nashville, Tennessee

**Keywords:** colitis, colon, ErbB4, macrophage, NRG4, TNF

## Abstract

Macrophages are critical mediators of intestinal defense and homeostasis. However, uncontrolled proinflammatory macrophage activity may contribute to chronic conditions such as inflammatory bowel disease. Currently, the regulatory feedback mechanisms restraining proinflammatory cytokine production in activated macrophages are not well understood. The ErbB4 receptor tyrosine kinase is induced on macrophages by proinflammatory stimulation, and chronic ErbB4 activation with its ligand NRG4 drives macrophage apoptosis after 2 days. However, the impact of endogenous NRG4/ErbB4 signaling on macrophage function remains untested. Using bone marrow-derived ErbB4-null or NRG4-null macrophages, we tested the hypothesis that NRG4/ErbB4 signaling inhibits proinflammatory cytokine production. We found that deletion of either the receptor or its ligand resulted in elevated proinflammatory cytokine expression in classically (IFNγ/LPS) activated cells, compared with activated cells generated from wild-type littermates. NRG4 was induced by IFNγ/LPS activation in wild-type macrophages, and exogenous treatment with NRG4 led to a reduction in *Tnf, Cxcl1,* and *Il1b* expression within 24 h. RNA sequencing of ErbB4^myeKO^ macrophages showed elevated expression of major regulators of inflammatory skewing (*Sik2*) and cytokine transport (*Trim16*). In vivo, ErbB4^myeKO^ mice subjected to acute DSS colitis showed exaggerated disease, and ErbB4 myeloid knockout in the IL 10-KO chronic colitis model accelerated disease onset. Taken together, these findings demonstrate that NRG4/ErbB4 signaling in macrophages restrains the proinflammatory tone of these cells and is an important limiting regulator of colitis severity. These results highlight a previously unknown feedback mechanism by which growth factor signaling in immune cells prevents runaway inflammation and chronic disease.

**NEW & NOTEWORTHY** Proinflammatory macrophages are essential drivers of colitis and express the growth factor receptor ErbB4. This study tested the role of ErbB4 and its specific ligand, NRG4, in regulating macrophage function. We show that endogenous NRG4-ErbB4 signaling limits macrophage production of proinflammatory cytokines in vitro and limits colitis severity in vivo and thus is a potential target for therapeutic intervention.

## Abstract (precis)

Proinflammatory macrophages are essential drivers of colitis and express the growth factor receptor ErbB4. This study tested the role of ErbB4 and its specific ligand, NRG4, in regulating macrophage function. We show that endogenous NRG4-ErbB4 signaling limits macrophage production of proinflammatory cytokines in vitro and limits colitis severity in vivo and thus is a potential target for therapeutic intervention.

## Graphical abstract

**Figure:**
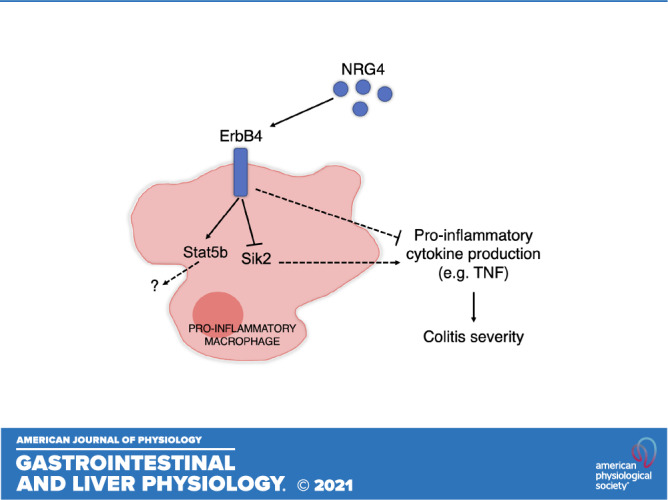

## INTRODUCTION

Inflammatory bowel disease (IBD) is a chronic illness affecting the gastrointestinal tract that presents with relapsing inflammatory injury. The underlying causes of IBD are not fully understood, but aggressive immune cell responses feature prominently (1). In particular, innate immune cells play a central role in initiating inflammation in the intestinal tract (2–4). Macrophages, for example, are recruited to sites of tissue injury where they release cytokines and chemokines that target potential pathogens while concurrently amplifying further immune activity (5). These cells exist along a continuum of functional states spanning from one extreme of anti-inflammatory activity that can also promote repair and tissue maintenance, to proinflammatory activation that mediates pathogen clearance (6). Activity of these cells, including cytokine production, at sites of injury is essential for preventing infection. However, their responses must also be appropriately limited as excessive inflammatory damage to tissue potentiates injury or induces chronic disease (7, 8).

ErbB receptor tyrosine kinases (EGFR, ErbB2, ErbB3, and ErbB4) are a class of growth factor receptors that are necessary for the maintenance of tissue homeostasis in the gut, and are dysregulated in IBD (9–13). Although they have historically been studied as epithelial trophic factors, recent work has shown that these receptors are also expressed on innate immune cell populations including macrophages, dendritic cells, and neutrophils. Several groups have identified anti-inflammatory effects of neuregulin (NRG)/ErbB signaling, with reported attenuation of inflammatory injury in heart (14, 15), lung (15), stomach (16), and colon (17, 18), although the precise mechanisms mediating these outcomes are not fully understood.

We previously reported that ErbB4 is specifically induced in proinflammatory (but not anti-inflammatory) macrophages (18). Treatment with its ligand NRG4 promotes macrophage apoptosis within 2 days and attenuates experimental colitis. However, the acute functional roles for endogenous ErbB4 signaling in macrophages and how these influence colitis have not been identified. To address this important knowledge gap, we used mice with a myeloid-specific deletion of ErbB4 and subjected them to in vitro and in vivo analysis. Here, we report that ErbB4 in macrophages suppresses proinflammatory cytokine production. Through RNA sequencing, we show that ErbB4 deletion in these cells drives a proinflammatory expression profile. Loss of macrophage ErbB4 exacerbated the severity of experimental colitis and promoted macrophage accumulation in tissue. Together, these results suggest an anti-inflammatory mechanism where ErbB4 signaling limits excessive cytokine production through feedback inhibition. Treatment with the ErbB4-specific ligand NRG4 can potentiate this effect and further limit cytokine production during resolution of inflammation. As inflammatory injury is a prominent feature of IBD and macrophages are central to potentiating disease severity, identifying ways to limit cytokine production in these cells may allow for the development of macrophage-specific targets to treat IBD.

## MATERIALS AND METHODS

### Animal Experiments

All animal use was approved and monitored by the Children's Hospital Los Angeles Institutional Animal Care and Use Committee (Animal Welfare Assurance A3276-01). Mice were housed under standard conditions with ad libitum water and chow access in the AAALAC-accredited animal care facility at Children’s Hospital Los Angeles. C57Bl/6J mice obtained from Jackson Laboratory aged 8–12 wk were used for experiments. Mice with a myeloid-specific deletion of ErbB4 (LysMCre/ErbB4^FF^; referred to as ErbB4^myeKO^) (15) were generated by crossing LysMCre animals (Strain 004781, Jackson Laboratory) with mice harboring LoxP-flanked ErbB4 (ErbB4^FF^) (19). ErbB4^myeKO^ and ErbB4^FF^ littermate controls were used for experiments. NRG4-KO mice were obtained from the Mutant Mouse Resource and Research Centers (Cat. No. MMRRC:011746-UCD) and back-crossed onto C57Bl/6 for >10 generations. Experimental mice were bred from NRG4-het parents to allow for NRG4-KO and NRG4-WT littermate controls. For acute colitis, mice were given 3% (wt/vol) dextran sodium sulfate (DSS, Affymetrix) in drinking water for 6 days (injury phase), followed by 6 days of DSS withdrawal (recovery phase). For chronic colitis, ErbB4^myeKO^ were mated to IL10-KO mice to generate mice that spontaneously develop immune-mediated colonic inflammation. Both male and female littermates were used for mouse experiments.

### Real-Time PCR

RNA from cells and tissue was collected using on-column RNA isolation and purification (Omega Bio-tek), and cDNA generated with a high-capacity cDNA reverse transcriptase kit (Applied Biosystems, 4368814). Quantitative analysis of expression was performed using TaqMan assays [*Hprt* (Mm03024075_m1), *Tnf* (Mm00443258_m1), *Il1b* (Mm00434228_m1), *Ifng* (Mm01168134_m1), *Cxcl1* (Mm04207460_m1), *Il4* (Mm00445259), Il6 (Mm00446190_m1), *Il10* (Mm01288386_m1), *Il12* (Mm00434169_m1), *Arg1* (Mm00475988_m1), and *Nrg4* (Mm00446254_m1)] on an Applied Biosystems StepOne Thermocycler. Fold change was calculated using the 2^−ΔΔCt^ method (20). Results are expressed as average fold change in gene expression relative to control or nontreatment group using HPRT as the reference gene.

### Immunohistochemical Staining

Colonic Swiss rolls were prepared from ErbB4^myeKO^ mice and ErbB4^FF^ littermate controls and fixed with 4% paraformaldehyde overnight. Sections (5 µm) were blocked with 10% goat serum for 1 h at room temperature and incubated with primary antibody against F4/80 (Caltag Laboratories, MF480-15) overnight at 4°C. Sections were then washed with DPBS, permeabilized with DPBS Triton X-100 (0.2%) for 1 h, and incubated with secondary goat anti-rat conjugated to HRP (Vector Laboratories) for 1 h at room temperature. Sections were incubated with diaminobenzidine (Vector Laboratories) followed by hematoxylin counterstain and Permount mounting.

### ELISA

Fecal lipocalin-2 levels in distal colon fecal contents were analyzed using the Mouse Lipocalin-2 DuoSet ELISA (R&D Systems, DY1857) according to manufacturer-provided instructions. TNF and CXCL1 levels from BM-Mɸ conditioned media were analyzed after 24-h activation using Mouse TNF DuoSet ELISA (R&D Systems, DY410) or Mouse CXCL1 DuoSet ELISA (R&D Systems, DY453). NRG4 levels in BM-Mɸs and epithelial isolates of colonic tissue were analyzed using the Mouse NRG4 ELISA Kit (Novus Biologicals, NBP2-76763).

### Bone Marrow-Derived Macrophages

Isolated bone marrow from mice was incubated with filtered CMG14–12 conditioned media (1:20) containing M-CSF to generate bone marrow-derived macrophages (BM-Mɸ) as previously described (21, 22). Adherent cells were washed at 3 days and refed with M-CSF containing media until experimentation at *days 7–8*. Cells were replated at a density of 100,000/mL. For M1 polarization, cells were pretreated with 100 U/mL IFNγ for 16 h and then stimulated with 100 ng/mL LPS from *Escherichia coli* 0111:B4, purified by gel-filtration chromatography (Sigma, St. Louis, MO; Cat. No. L3012). For M2 polarization, cells were stimulated with 20 ng/mL IL-4 (Gibco, Waltham, MA; PMC0046). BM-Mɸs were collected at the time point indicated for each experiment post-LPS or IL-4 treatment. For some experiments, cells were incubated with 100 ng/mL NRG4 (Reprokine) for the time indicated in respective experiments.

### RNA Sequencing and Analysis

RNA isolated from M1-activated macrophages (from 6 ErbB4^myeKO^ and 6 ErbB4^FF^ littermates) was barcoded using Illumina index primers and sequenced on a NextSeq machine to obtain single-ended reads of 75 bp length to a depth of ∼10 M reads/sample. Data are publicly available through the NCBI Gene Expression Omnibus (GSE168070). FASTQ files were pseudoaligned using kallisto (23) to the mouse transcriptome to obtain transcript per million (tpm) abundance estimates. Tests for genotype-driven differential expression were performed in sleuth (24) and were linearly adjusted for litter and sex covariates. To compare pathway enrichment, transcript abundances were summed to obtain a per-gene expression value, which was then inputted into GSEA (25). Enrichment of interferon pathways was identified using the “Hallmark” database of targets (26). Specific tests for enrichment of M1 macrophage signals were performed using the GSEA algorithm against an M1 gene set profile containing gene symbols: CCL20, CCL5, CCR7, CXCL10, CXCL13, CXCL2, CXCL3, IFNG, IL12A, IL12B, IL1A, IL23A, IL27, IL6, IRF5, PTGS2, SOCS3, and TNF. Plots were generated in R.

### Western Blot Analysis

Cellular proteins from activated macrophages were isolated in modified RIPA buffer (11) containing Halt Protease Inhibitor Cocktail (Thermo Scientific, 1861278) and phosphatase inhibitor cocktails (Sigma, P5726 and P0044). Protein concentration was determined by DC assay (Bio-Rad, 500). Ten micrograms of protein/condition was separated by SDS-PAGE (Thermo Scientific, NW0412A) and transferred to nitrocellulose membranes, which were blocked with 5% nonfat powdered milk in Tris-buffered saline plus 0.1% Tween-20 and probed with rabbit anti-Stat5b (1:1,000; Cell Signaling, 34662) or mouse anti-actin (1:10,000; Sigma, A1978) overnight at 4°C, followed by washing and HRP-conjugated secondary antibodies (1:2,500; Cell Signaling 7074 and 7076) for 1 h at room temperature. Labeled blots were washed, incubated with ECL detection reagent (Thermo Fisher, 32132), scanned on a C-Digit (LI-COR), and quantitated in ImageStudio (LI-COR) software.

### Statistical Methods

Statistical analyses and plots were generated using Prism (GraphPad Software). Means ± SE is depicted in bar graphs. Student’s *t* test or ANOVA with Tukey post hoc test to correct for multiple comparisons was used to determine statistical differences, as appropriate. Statistical significance was assigned to *P* < 0.05.

## RESULTS

### ErbB4 Deletion in Macrophages Results in Elevated Proinflammatory Cytokine Production in Vitro

Previous studies showed that neuregulin/ErbB signaling in macrophages can reduce tissue inflammation in intestine, heart, and lung (14, 15, 18). However, the receptor specificity and cellular mechanisms of these effects are not fully understood. We have previously shown that sustained treatment of proinflammatory macrophages with the ErbB4-specific ligand NRG4 induces apoptosis over time (18). However, the acute regulatory role of ErbB4 in these cells has yet to be tested. To understand the impact of ErbB4 on cytokine production, we generated bone marrow-derived macrophages (BM-Mɸs) from mice with a myeloid-specific deletion of ErbB4 (LysMCre/ErbB4^FF^; referred to as ErbB4^myeKO^) (15) and ErbB4^FF^ littermate controls. BM-Mɸs were stimulated with interferon (IFN-γ) and lipopolysaccharide (LPS) (so-called “M1” activation, an artificial but experimentally useful classification) or interleukin (IL)-4 (“M2” activation) for 6 h. M1-activated macrophages rapidly produce inflammatory cytokines following sensing of LPS by toll-like receptor 4 on the cell surface.

**Figure 1.**
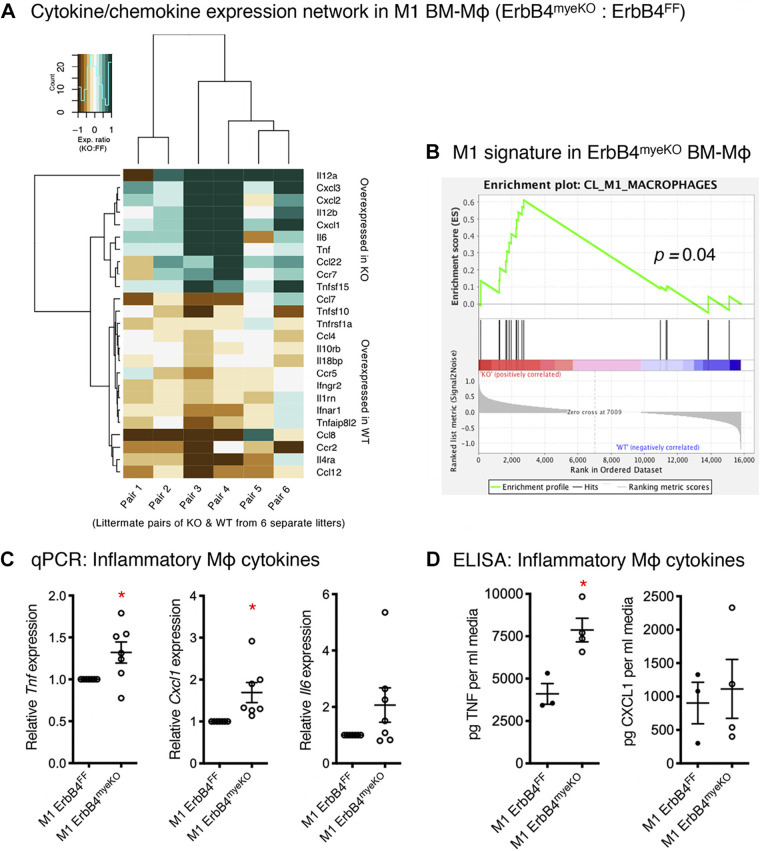
ErbB4 deletion results in enhanced inflammatory profile of activated BM-Mɸs. RNA sequencing on classically “M1”-activated WT and ErbB4-null bone marrow-derived macrophages (BM-Mɸs) was performed. *A*: hierarchical clustering of inflammatory mediators showed distinct changes in patterns of cytokine and chemokine expression networks. Data are generated as relative expression from pairs of ErbB4^myeKO^ and ErbB4^FF^ littermates from six separate litters. *B*: gene set enrichment analysis (GSEA) for M1 markers showed a significant enrichment in proinflammatory gene expression in ErbB4^myeKO^ BM-Mɸs compared with ErbB4^FF^ littermate BM-Mɸs at 6 h postactivation. *C* and *D*: expression of key inflammatory cytokines was assessed by qPCR (*n* = cultures from 7 mice/group) and ELISA (*n* = cultures from 3 or 4 mice/group). *P < 0.05.

To assess the overall effects of ErbB4 loss on cytokine and chemokine production, we performed bulk RNA sequencing on M1-activated BM-Mɸ generated from ErbB4^myeKO^ mice and ErbB4^FF^ littermate controls. Hierarchical clustering of a panel of cytokines, cytokine receptors, and binding proteins shows enhanced expression of M1-associated cytokines (Fig. 1*A*), with reduced expression of others. Notably, classically activated ErbB4-deficient macrophages showed enriched expression of the proinflammatory cytokines IL-12, TNF, chemokine (C-X-C motif) ligand (CXCL) 1–3, and IL-6, but diminished expression of chemokine (C-C motif) ligand (CCL) 7 and CCL12, suggesting a specificity to the pattern of cytokine regulation within M1 cells. We also detected reduced expression of TNF-related apoptosis-inducing ligand (TRAIL, a.k.a. Tnfsf10) which is involved in apoptosis and macrophage regulation (27). Gene set enrichment analysis (GSEA) showed significant enrichment of an M1-associated profile (Fig. 1*B*).

**Figure 2.**
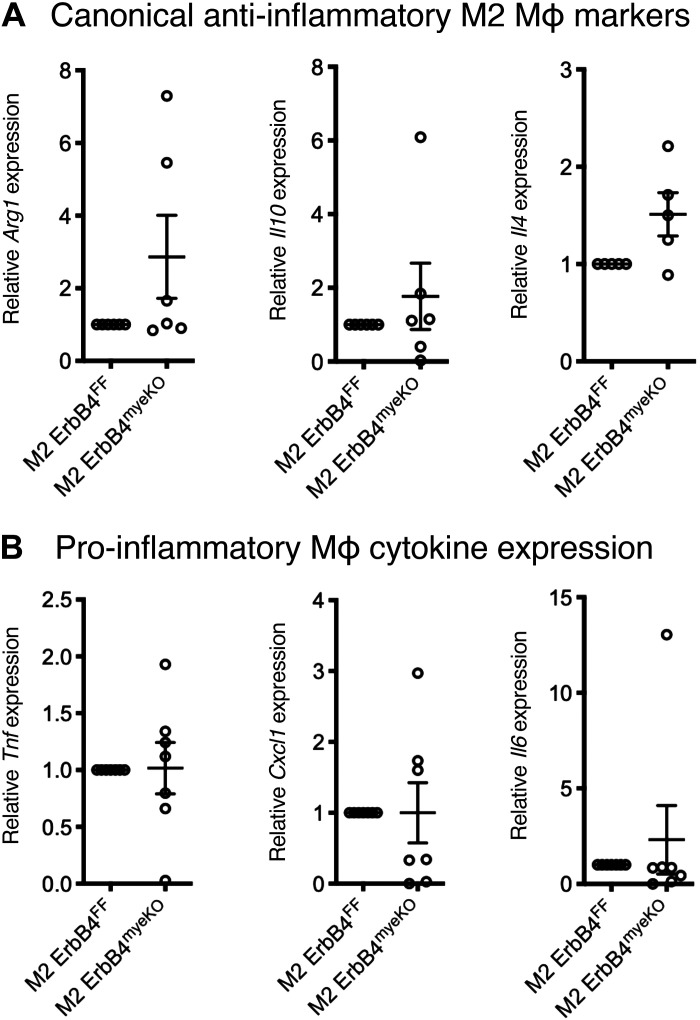
Cytokine production is not altered in “M2” anti-inflammatory BM-Mɸs. BM-Mɸs generated from ErbB4^myeKO^ and ErbB4^FF^ littermates were polarized to an anti-inflammatory M2 state with 20 ng/mL IL-4 for 6 h. Canonical M2 gene expression (*A*) and canonical M1 gene expression (*B*) were assessed by qPCR. *n* = cultures from 4 or 7 mice/group.

To further confirm these findings, we assayed for several classical M1 cytokines by qPCR and found significant inductions of *Tnf* and *Cxcl1*, indicating a higher level of proinflammatory induction within these cells (Fig. 1*C*). To determine whether elevated cytokine expression translated to an increase in secreted cytokines, we performed ELISAs for TNF and CXCL1 on conditioned media from mice. This analysis confirmed elevated TNF production in M1-activated BM-Mɸ generated from ErbB4^myeKO^ mice (Fig. 1*D*). However, increased CXCL1 levels were not observed, suggesting an additional layer of regulation may exist for secretion of this cytokine (Fig. 1*D*). ErbB4 is not expressed on M2-polarized BM-Mɸ (18); as predicted given this observation, analysis of canonical M2 genes, *Arg1*, *Il10*, and *Il4*, in M2-activated macrophages showed no difference between ErbB4^FF^ and ErbB4^myeKO^ BM-Mɸ (Fig. 2*A*). Likewise, in M2-polarized BM-Mɸ from ErbB4^myeKO^ mice, no changes were detected in proinflammatory cytokines *Tnf*, *Cxcl1*, and *Il6*, supporting the finding that ErbB4 activity is restricted to proinflammatory cells (Fig. 2*B*).

### NRG4 Is Induced in Classically Activated BM-Mɸ as an Autocrine Feedback Loop

**Figure 3.**
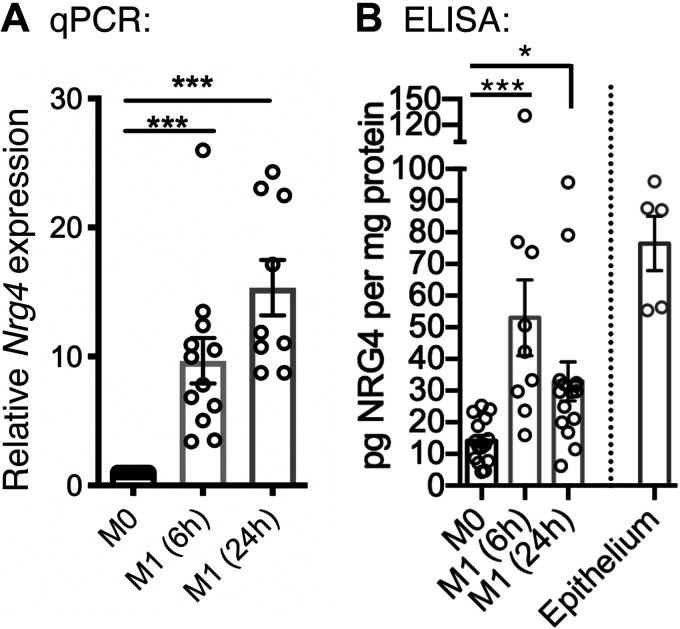
The ErbB4-specific ligand NRG4 is expressed by proinflammatory macrophages. Wild-type BM-Mɸs were activated with IFN-ɣ/LPS; RNA and epithelial isolates were collected at 6 h and 24 h poststimulation. Nrg4 expression was determined by qPCR (*A*) and ELISA (*B*) in BM-Mɸs (*n* = cultures from 9 or 15 mice/group) and epithelial isolates (*n* = 5 mice). *P < 0.05; ***P < 0.001.

ErbB4 is induced following proinflammatory activation in macrophages (18). However, the source of NRG4, its selective ligand, has been unclear. In intestinal epithelial cells, inflammatory stimulation results in a loss of NRG4 expression (28). To test its expression in macrophages, we analyzed M1-activated BM-Mɸ and found significant upregulation of NRG4 mRNA and protein, suggesting a potential autocrine feedback mechanism (Fig. 3*, A* and *B*). In colonic epithelial isolates, a comparable level of NRG4 expression level was observed when compared with activated BM-Mɸ (Fig. 3*B*).

**Figure 4.**
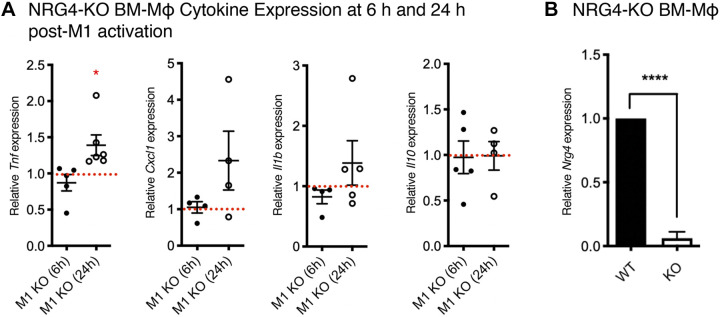
Endogenous neuregulin-4 limits proinflammatory cytokine production in M1 macrophages. Inflammatory gene expression was analyzed by qPCR in BM-Mɸs generated from NRG4-KO mice and wild-type littermates at 6 and 24 h poststimulation. Data are presented relative to wild-type littermates at each respective time point normalized to 1 (indicated by the dashed red line). *A*: at 6 h post-M1 activation, no cytokines are significantly altered in NRG4-KO BM-Mɸs. At 24 h post-M1 activation, *Tnf* expression is significantly elevated. *B*: NRG4 knockout was confirmed in BM-Mɸs by qPCR. *n* = cultures from 4 or 6 mice/genotype, from four separate litters; *P < 0.05; ****P < 0.0001.

To test whether endogenous NRG4 on inflammatory macrophages regulates function, we generated BM-Mɸ from NRG4-KO mice and activated with IFNγ/LPS. As NRG4 is produced as a membrane-bound protein and undergoes proteolytic processing to be shed and potentially regulate activity (29), we analyzed cytokine production in BM-Mɸ acutely at 6 h postactivation and 24 h postactivation. At 6 h, we found no difference in expression of the proinflammatory cytokines *Tnf*, *Cxcl1, or Il1b* by qPCR (Fig. 4*A*). However, by 24 h, we observed a significant increase in *Tnf* expression in NRG4-KO cells relative to wild type (Fig. 4*A*). The anti-inflammatory cytokine *Il10* was not altered at either time point (Fig. 4*A*). These findings suggest that endogenous NRG4 produced by macrophages limits proinflammatory cytokine expression as a negative feedback loop. In wild-type M1-activated macrophages, increased NRG4 levels are sustained over 24 h of activation (Fig. 3), consistent with the idea that ongoing synthesis of NRG4 is needed to achieve a threshold level for suppression of cytokine production.

### Exogenous NRG4 Treatment Limits Proinflammatory Cytokine Production

**Figure 5.**
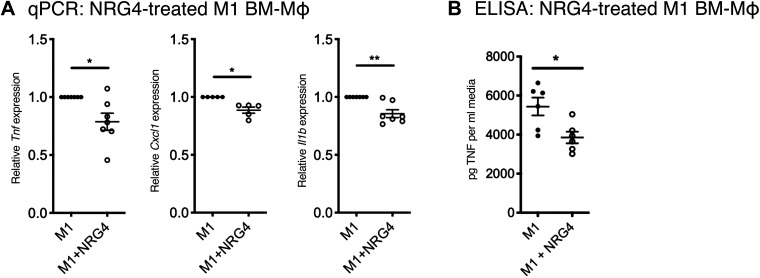
Exogenous neuregulin-4 reduces BM-Mɸ inflammatory cytokine expression. M1 BM-Mɸs derived from C57Bl/6 mice were treated with 100 ng/mL NRG4 for 24 h. *A*: expression of inflammatory cytokines *Tnf, Cxcl1*, and *Il1b* was assessed by qPCR. *B*: TNF in media was measured by ELISA. *n* = cultures from 5 or 7 mice/group. *P < 0.05; **P < 0.01.

Since endogenous NRG4 limits inflammatory cytokine production (Fig. 4), we hypothesized that pharmacological NRG4 treatment will do so as well. To test this, we treated M1-activated wild-type murine BM-Mɸ with recombinant NRG4 (100 ng/mL) for 24 h, followed by qPCR analysis. This concentration of NRG4 has been previously shown to induce optimal ErbB4 phosphorylation in vitro (30) and elicits functional effects in mouse and human macrophage cultures (18). NRG4 provoked a significant decrease in *Tnf* expression (Fig. 5*A*), supporting that the effects observed in NRG4-KO BM-Mɸ may be due to acute ligand-dependent effects. We also observed a significant decrease in *Cxcl1* and *Il1b*, consistent with a generalized reduction in proinflammatory cytokines with exogenous NRG4 treatment (Fig. 5*A*). As TNF is a major driver of disease in IBD and is secreted at elevated levels from ErbB4^myeKO^ BM-Mɸ, we measured TNF secretion in media from NRG4-treated M1 BM-Mɸ and found reduced levels consistent with reduced RNA levels (Fig. 5*B*).

### ErbB4 Deletion from Macrophages Exacerbates Experimental Colitis

We previously showed that exogenous NRG4 has anti-inflammatory activity in experimental colitis (17, 18). However, multiple tissues in the colon express the NRG4 receptor, ErbB4, thus making the direct role of ErbB4 in macrophages during colitis unclear.

**Figure 6.**
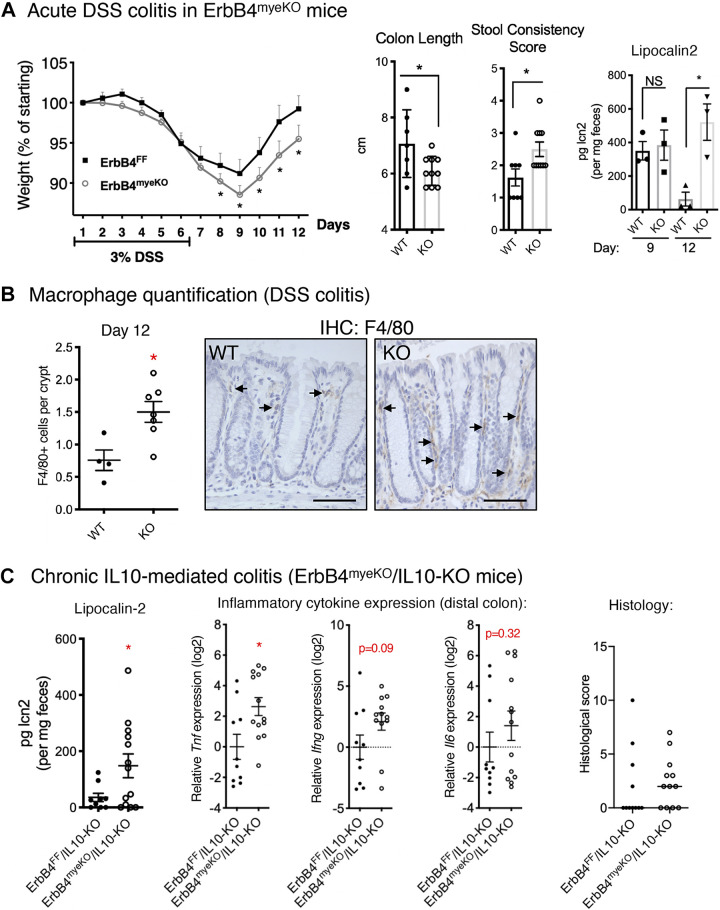
Loss of ErbB4 in macrophages exacerbates acute and chronic models of colitis. *A*: ErbB4^myeKO^ and littermate controls (ErbB4^FF^) were subjected to an acute model of DSS colitis: 3% DSS in drinking water for 6 days followed by 6 days without DSS. Weights of mice were recorded daily; *n* = 12 or 14 mice/group. On *day 12*, colon shortening and stool consistency were measured. To assess clinical severity following recovery, fecal lipocalin-2 content was measured on *day 12*; *n* = 3 mice per group. *B*: colonic sections were stained for the macrophage marker, F4/80, and positive cells were counted within the distal most 100 crypts, as inflammation is most pronounced in the distal region in DSS colitis. WT, ErbB4^FF^; KO, ErbB4^myeKO^. *C*: measures of inflammation are elevated in a chronic immune-mediated experimental colitis model. ErbB4^myeKO^/IL10-KO and littermate ErbB4^FF^/IL10-KO mice were analyzed at 8 wk of age for colitis onset. To assess severity of inflammation, fecal lipocalin-2 content was measured. Expression of inflammatory markers in distal colonic homogenate was analyzed by qPCR and presented as log2-fold changes. Colonic sections were scored for colitis (integrated score of enterocyte loss, hyperplasia, crypt inflammation, and immune cell infiltrate). *n* = 10 or 13 mice/group. *P < 0.05. DSS, dextran sodium sulfate.

To test the impact of loss of NRG4/ErbB4 signaling specifically in macrophages during colitis, we used ErbB4^myeKO^ mice. Within immune cells, induction of ErbB4 by challenge is exclusive to proinflammatory macrophages (18); thus, this is functionally a specific knockout for these cells. ErbB4^myeKO^ and ErbB4^FF^ littermate controls were subjected to an acute model of dextran sodium sulfate (DSS) colitis by administration of 3% DSS in drinking water for 6 days to induce injury, followed by 6 days of standard drinking water to allow for recovery. ErbB4^myeKO^ mice lost more weight indicating a more severe course of colitis (Fig. 6*A*). These mice also showed reduced colon length and looser stool consistency after 12 days (Fig. 6*A*). Analysis of fecal lipocalin-2 levels (a sensitive clinical marker of intestinal inflammation (31)) showed that although severity of inflammation was similar at *day 9*, colitis persisted in ErbB4^myeKO^ mice through *day 12* (Fig. 6*A*). Furthermore, in accordance with previous findings showing that ErbB4 signaling promotes macrophage apoptosis, we found elevated numbers of macrophages in ErbB4^myeKO^ colons (Fig. 6*B*).

To determine the impact of loss of macrophage ErbB4 in a chronic model of immune-mediated colitis where macrophages are a key driver of colitis initiation (2, 3), ErbB4^myeKO^ mice were crossed with IL 10-KO mice, which develop colitis spontaneously. Colons from ErbB4^myeKO^/IL10-KO mice collected at 8 wk of age displayed elevated markers of inflammation onset, including elevated fecal lipocalin-2 and increased *Tnf* expression compared with IL 10-KO littermates (Fig. 6*C*). Expression of *Ifng*, a cytokine involved in mediating colitis in the IL 10-KO model, trended toward increased expression, although this did not meet statistical significance, and expression of *Il6*, a cytokine with both pro- and anti-inflammatory effects in colitis, was not significantly elevated (32). Furthermore, more double knockout mice developed histologically detectable disease by 8 wk (67% of ErbB4^myeKO^/IL 10-KO mice vs. 36% of IL 10-KO littermates), although at this age the mean histological scores of the two groups were not yet significantly different, suggesting that loss of macrophage ErbB4 is acting in the early stages of disease onset.

### Global Analysis of Gene Expression in ErbB4-Deleted M1 Macrophages

**Figure 7.**
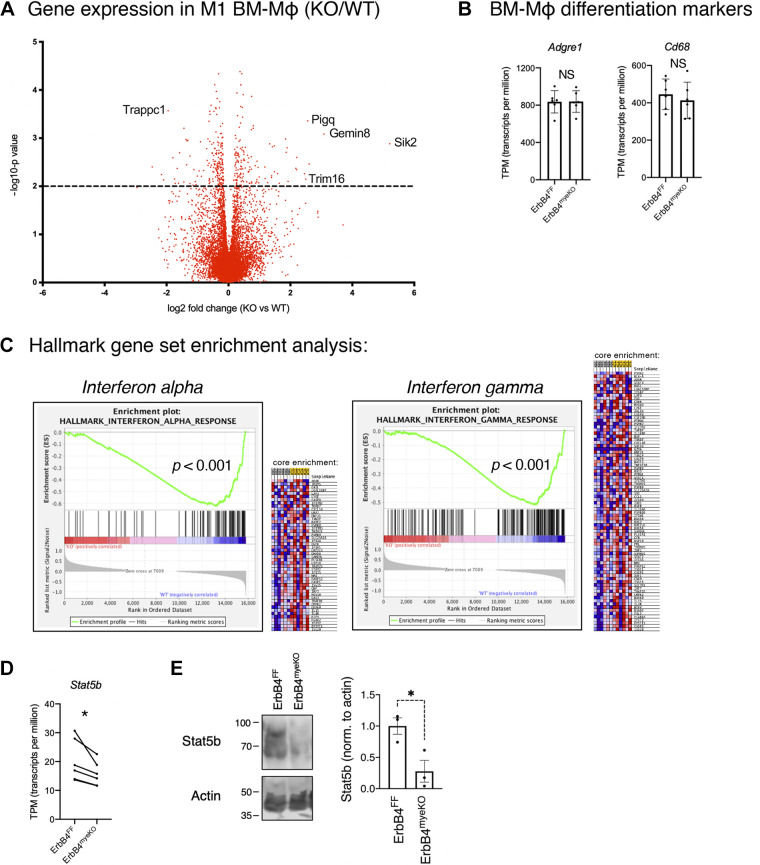
Global gene analysis of ErbB4^myeKO^ M1 BM-Mɸs. Analysis of RNA sequencing on proinflammatory BM-Mɸs generated from ErbB4^myeKO^ and ErbB4^FF^ mice. *A*: volcano plot of genes (at *P* < 0.01) regulated in M1-activated ErbB4^myeKO^ BM-Mɸs (each plotted point indicates the average log_2_ expression of ErbB4^myeKO^ BM-Mɸ compared with ErbB4^FF^ BM-Mɸs). *B*: markers of macrophage differentiation are not altered in ErbB4^myeKO^ BM-Mɸs. *C*: gene set enrichment analysis on hallmark gene sets determined significant loss of interferon α and interferon γ signatures in ErbB4^myeKO^ BM-Mɸs. *D*: Stat5b expression in M1 BM-Mɸs from paired ErbB4^myeKO^ and ErbB4^FF^ littermates. *n* = cultures from six mice per group, litter matched from six separate litters. *E*: Western blot for Stat5b and actin in M1 BM-Mɸs. *n* = cultures from 3 mice/group. *P < 0.05.

To understand the pathways by which ErbB4 may be regulating proinflammatory cytokine production, we returned to an analysis of global gene regulation with our RNA sequencing of M1-activated BM-Mɸ generated from ErbB4^myeKO^ mice and ErbB4^FF^ littermates (Fig. 7). We confirmed in this data set that expression of the macrophage markers *Adgre1* and *CD68* was unchanged, demonstrating that the deletion of ErbB4 did not alter the M-CSF-directed developmental programming of bone marrow progenitors into macrophages (33, 34) (Fig. 7*B*). Next, we identified a number of altered genes and pathways associated with regulating the inflammatory state of macrophages and cytokine release. Interestingly, the highest elevation in expression in ErbB4-null macrophages was in the gene *Sik2*, which codes for a member of the AMP-activated protein kinase (AMPK) family thought to act as a regulator of metabolic processes. Downregulation of this gene enhances anti-inflammatory tone and limits *Tnf* production; thus, an elevated expression of *Sik2* may contribute to the increased proinflammatory tone we observe (35, 36). We also found increased expression of *Trim16*, which codes for a protein responsible for IL-1β processing and release (37), and reduced expression of *Trappc1*, a protein transporter elevated in LPS-activated macrophages (38). These findings suggest ErbB4 may directly or indirectly regulate expression of these individual genes leading to reduced proinflammatory activation and altered cytokine expression in activated macrophages.

To gain a global understanding of pathways regulated by ErbB4, we performed GSEA between ErbB4^myeKO^ and ErbB4^FF^ for hallmark gene sets. Notably, this analysis revealed that loss of type I interferon (α) and type II interferon (γ) signaling in ErbB4^myeKO^ BM-Mɸs were the most significantly altered pathways (Fig. 7*C*). ErbB4 directly promotes Stat activation, a regulator of interferon signaling, in some model systems (39, 40), and our analysis showed reduced Stat5b expression at both the mRNA and protein levels in ErbB4^myeKO^ BM-Mɸs (Fig. 7, *D* and *E*). Thus, our findings indicate a potential role for ErbB4 in macrophages in coordinating interferon signaling via Stat regulation.

## DISCUSSION

Rapid and aggressive macrophage recruitment and activation following damage or infection in the gut is essential for maintaining tissue health and integrity. However, an overabundance of immune cell recruitment with sustained activity can result in deleterious outcomes such as tissue ulceration, loss of barrier function, and a compromised stem cell niche (41–43). Inflammatory bowel disease (IBD) is characterized by elevated levels of proinflammatory cytokines in the gastrointestinal tract, and excessive cytokine production in intestinal macrophages is a likely contributor to the pathogenesis of IBD (44, 45).

Little is known about the mechanisms restraining macrophage activity once cells have been activated by environmental stimuli (e.g., bacterial LPS and TNF). The most well-studied negative regulator of macrophage inflammatory activation is the anti-inflammatory cytokine, IL-10. Intestinal resident tissue macrophages express high levels of the IL-10 receptor, and deletion of this receptor induces spontaneous colitis (46). Once inflammatory stimuli (e.g., pathogenic bacterial LPS) overcome the activation threshold of intestinal macrophages, they polarize to a proinflammatory state and produce a wave of cytokines to expand the immune response and promote pathogen removal. Excessive cytokine levels are cytotoxic and deleterious to the host, and thus, this response must be tempered in duration and magnitude, but the mechanisms that mediate this regulation are not fully understood (47).

We have previously demonstrated that ErbB4 is induced selectively on proinflammatory (“M1”)-activated macrophages, and sustained incubation with its ligand, NRG4, promotes inflammatory macrophage apoptosis (18). As such, ErbB4 signaling can limit the accumulation of immune cells at sites of infection in the gut. In this work, we extended these findings to understand the role of endogenous NRG4/ErbB4 signaling in a central functional activity of macrophages—cytokine production—through the use of ErbB4-null and NRG4-null models. We report that, before it eventually triggers apoptosis, NRG4/ErbB4 also plays a substantial role in restraining production of key cytokines in proinflammatory macrophages.

Interestingly, we found that the ErbB4-specific ligand, NRG4, is highly induced in M1-activated macrophages. Previous reports showed it is expressed in epithelial and mesenchymal tissues; however, no studies to date have demonstrated expression in immune cells. To understand the role of this ligand on macrophage biology, we tested the effects of both exogenous NRG4 treatment and endogenous NRG4 deletion. Similar to the previous work showing that NRG1 (a nonspecific ligand that can bind both ErbB3 and ErbB4 and activate any of the ErbBs depending on context) limits cytokine production in monocytes (14, 15), we found that acute NRG4 treatment led to a significant reduction in *Tnf*, *Cxcl1*, and *Il1b*. Though the magnitude of these changes was moderate, a sustained moderate shift in cytokine production from macrophages may contribute to disease development in IBD (48). The fact that both NRG1 and NRG4 are capable of reducing cytokine production reinforces the hypothesis that ErbB4 [the only receptor activated by both (49)] signaling in particular controls the inflammatory tone of macrophages. Some ErbB family members such as EGFR can actually enhance inflammatory activity of macrophages or inhibit anti-inflammatory “M2” polarization (16, 50, 51) instead of inhibiting proinflammatory activity as described here for ErbB4. Thus, ErbB signaling may control a network of positive and negative pathways regulating macrophage polarization and function. These opposing functions within the same receptor family underscore the need to understand the role of individual receptors and ligands in these processes.

After inflammatory stimulation, NRG4-null macrophages expressed significantly higher levels of TNF, which suggests that an autocrine feedback loop may serve to prevent overaggressive cytokine responses. Colonic NRG4 is lost in IBD (17) and may reflect a dysregulated NRG4/ErbB4 feedback loop in disease. Thus, macrophage-expressed NRG4 may represent an attempt to compensate for loss of epithelial NRG4 or could be a regulatory mechanism by which the action and target of signaling by this growth factor are localized to specific microenvironments. Since NRG4 can be secreted (52), it will likely act both as an autocrine signal and in a paracrine fashion on locally recruited cells. We speculate this may be a population mechanism to limit excessive inflammatory activation when large numbers of macrophages have been recruited to tissue—a sort of “quorum sensing” but for inflammatory activity. This concept and the signals regulating NRG4 secretion will need to be explored further in subsequent work.

RNA sequencing of M1-activated ErbB4-null BM-Mɸs revealed an overall enhanced inflammatory tone and identified regulation of genes noted for their ability to modulate cytokine production or release. Multiple networks control cytokine production, processing, and release in macrophages. Notably, we identified genes that control IL-10 production (*Sik2*) and genes that regulate cytokine processing and release (*Trim16* and *Trappc1*). These will be important targets for future study in determining if these are directly modulated by loss of ErbB4 or a functional consequence of elevated inflammatory cytokines produced in ErbB4-null macrophages. We also found that ErbB4^myeKO^ macrophages showed reduced expression of *Tnfsf10,* which encodes TRAIL, a well-studied mediator of cellular apoptosis. This suggests that this clearance mechanism may be dysregulated in the absence of ErbB4, thus accounting for the elevated numbers of macrophages observed in DSS experiments (Fig. 6*B*). Another intriguing finding was the loss of type I and type II interferon signatures in ErbB4-null macrophages by GSEA. This is notable as ErbB4 signaling has previously been linked to Stat1 and Stat5 activation (39), both of which act as central regulators of interferon signaling (Stat1: type I and II; Stat5: type I) (53, 54). Roughly, type I interferon (α, β) signaling provides antiviral responses that can limit proinflammatory cytokine production, whereas type II interferon (γ) signaling can promote proinflammatory pathways. It is conceivable that ErbB4, through its interaction with Stat1 and Stat5, provides a checkpoint to modulate interferon signaling, and a balance between type I and type II signaling underlies the phenotypic observations. Future work will need to test the prospect that ErbB4 regulates Stat/IFN signaling in macrophages and address the impact on regulation of cytokine production and apoptosis. It is important to note, however, that studies to date connecting ErbB4 and Stat were performed in nonimmune cells. Thus, further work is necessary to determine whether this mechanism is conserved in macrophages. Taken together, these findings suggest that ErbB4 may control a number of switches within the cell to elicit its effects on cytokine production and macrophage persistence. However, whether this is causal to the enhanced proinflammatory tone of ErbB4^myeKO^ macrophages or a side effect of ErbB4 directly regulating cytokine production remains to be determined.

In summary, our data demonstrate that ErbB4 acts as a regulator of cytokine production in proinflammatory-activated macrophages. The neuregulin/ErbB family limits inflammation in several animal models, and we have previously found that NRG4 limits the severity of acute colitis. This study extends these findings to identify the specific role of acute NRG4/ErbB4 signaling in macrophages and the impact that loss of ErbB4 in macrophages has on acute and chronic colitis. As the neuregulin/ErbB family is dysregulated in IBD, this points to a novel way in which unrestrained signaling may perpetuate inflammation. We have identified a mechanism by which local growth factors limit macrophage activity and thus restrain overaggressive inflammatory responses. Loss of signaling through this receptor may promote an elevated and prolonged inflammatory state in the face of disease. Identifying ways to target this signaling may aid in limiting intestinal inflammation.

## GRANTS

This work was supported by funding from the National Institutes of Health (Grant No. R01DK095004 to M. Frey) and the Crohn’s & Colitis Foundation (Senior Research Award to M. Frey; Research Fellowship Award and Career Development Award to M. Schumacher).

## DISCLOSURES

M. R. Frey and M. A. Schumacher are inventors on a patent held by Children’s Hospital Los Angeles on the possible therapeutic use of NRG4 in intestinal inflammation. None of the other authors has any conflicts of interest, financial or otherwise, to disclose.

## AUTHOR CONTRIBUTIONS

M.A.S. and M.R.F. conceived and designed research; M.A.S., I.C.D., C.R., and J.S. performed experiments; M.A.S., I.C.D., C.Y.L., C.R., M.K.W., and M.R.F. analyzed data; M.A.S., I.C.D., C.Y.L., C.R., and M.R.F. interpreted results of experiments; M.A.S., I.C.D., C.Y.L., and C.R. prepared figures; M.A.S. and M.R.F. drafted manuscript; M.A.S., I.C.D., C.Y.L., J.S., J.K.B., M.K.W., D.B.P., and M.R.F. edited and revised manuscript; M.A.S., I.C.D., C.Y.L., C.R., J.S., J.K.B., D.B.P., and M.R.F. approved final version of manuscript.
